# Supplementary figures and images for: Characterization of Unique Pathological Features of COVID-Associated Coagulopathy: Studies with AC70 hACE2 Transgenic Mice Highly Permissive to SARS-CoV-2 Infection
Source: PLoS Pathog. 2024 Jun 24;20(6):e1011777. doi: 10.1371/journal.ppat.1011777 (PMC11226087; doi:10.1371/journal.ppat.1011777)

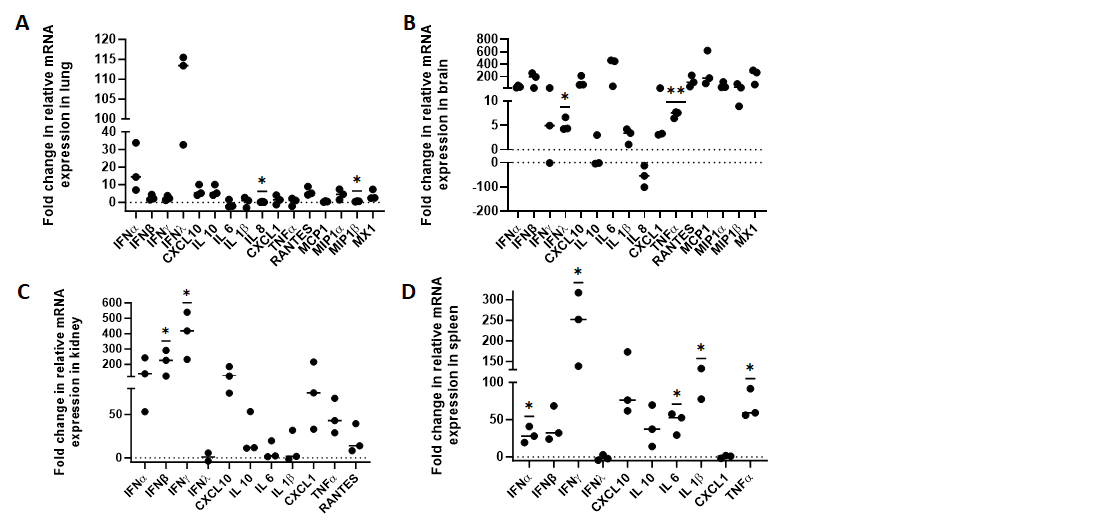

Supplement: S1 Fig — AC70 Tg+ mice were challenged intranasally with 105 TCID50 of SARS-CoV-2 and sacrificed at 4 d.p.i. Total RNA was isolated from (A) lungs, (B) brains, (C) kidneys, (D) spleens, and cytokines and chemokines expressions were quantified by RT-qPCR. Results are shown as mean relative fold change in expression compared to Tg- mice and were normalized to 18S RNA. *p < 0.05, **p < 0.01 Tg+AC70 vs Tg-. (TIF) [file ppat.1011777.s001.tif]

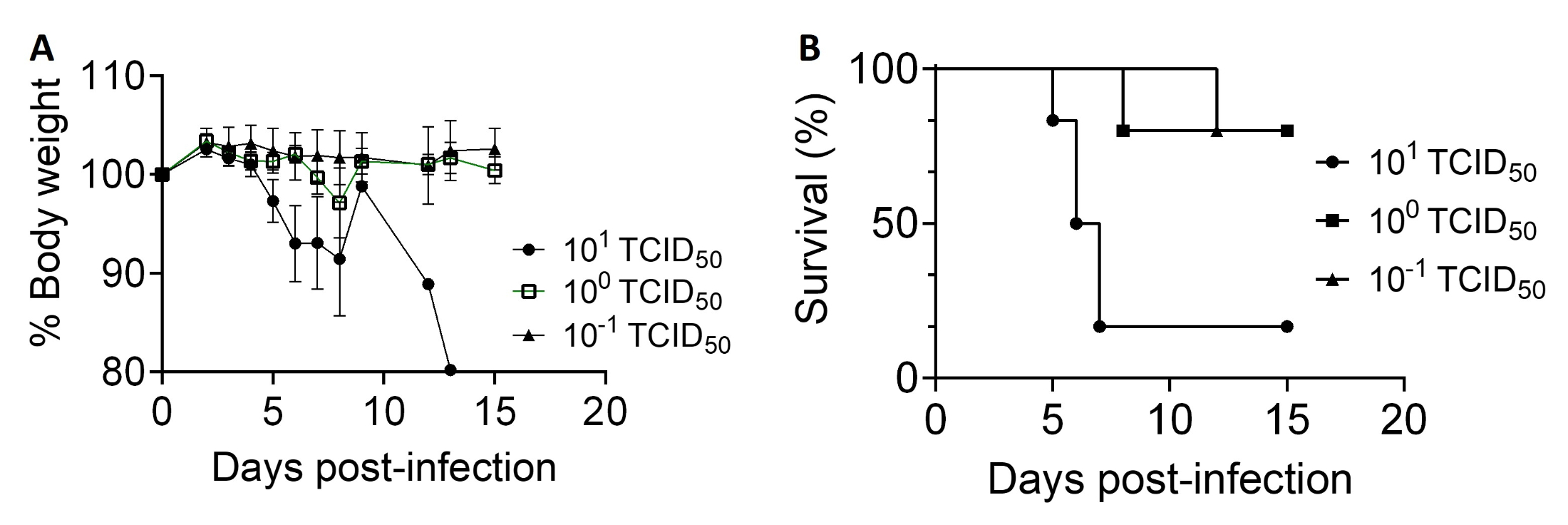

Supplement: S2 Fig — Mice (n = 6) were challenged with 1 × 101, 1 × 100 and 1 × 10^−1^ TCID50 of SARS-CoV-2. (A) % body weight change, and (B) % survival. Estimated LD50 is ~3. (TIF) [file ppat.1011777.s002.tif]

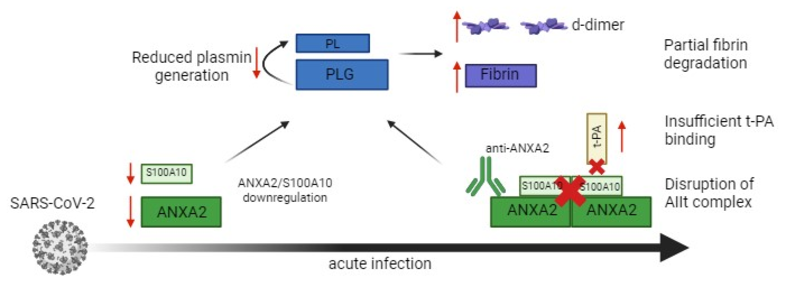

Supplement: S3 Fig — Acute SARS-CoV-2 infection promptly down-regulated the transcriptional expressions of Annexin A2 (ANXA2), and S100A10-encoding gene, the binding partner of ANXA2. Additionally, the early detection of ANXA2-specific autoantibody by infected mice might add to the disturbance the formation of the ANXA2/ S100A10 complex (AIIt) formation, which subsequently impaired the binding of t-PA to its cellular receptor, thereby affecting its biological function. Dysregulation of ANXA2 mechanisms may lead to suppression of plasmin generation even in the presence of abundant t-PA, resulting in insufficient fibrin degradation evidenced by increase d-dimer levels and fibrin depositions. PLG: plasminogen, PL: plasmin. (TIF) [file ppat.1011777.s003.tif]

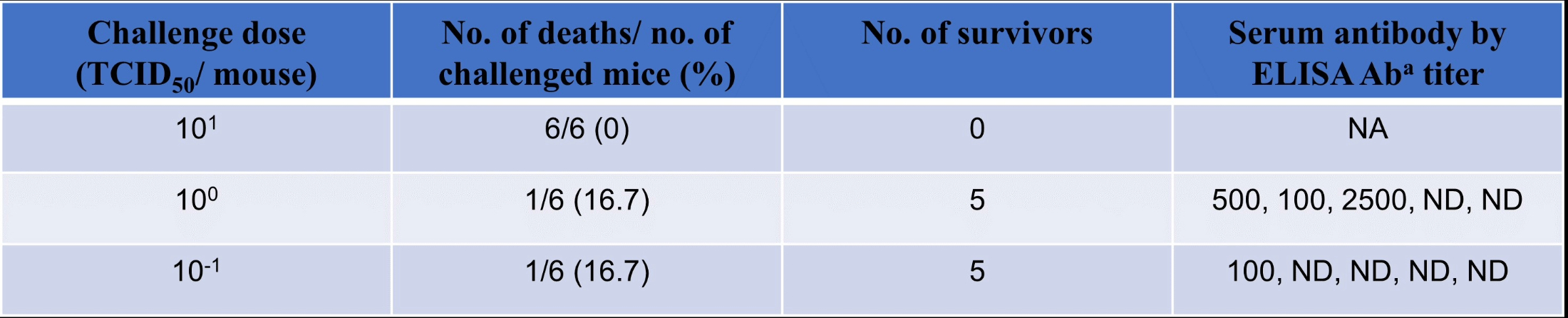

Supplement: S1 Table — SARS-CoV-2 antibody response was determined by analysis of serum specimens from survived mice at 21 d.p.i. ND: not detected; NA: not applicable. Estimated ID50 is ~0.51 TCID50. (TIF) [file ppat.1011777.s004.tif]

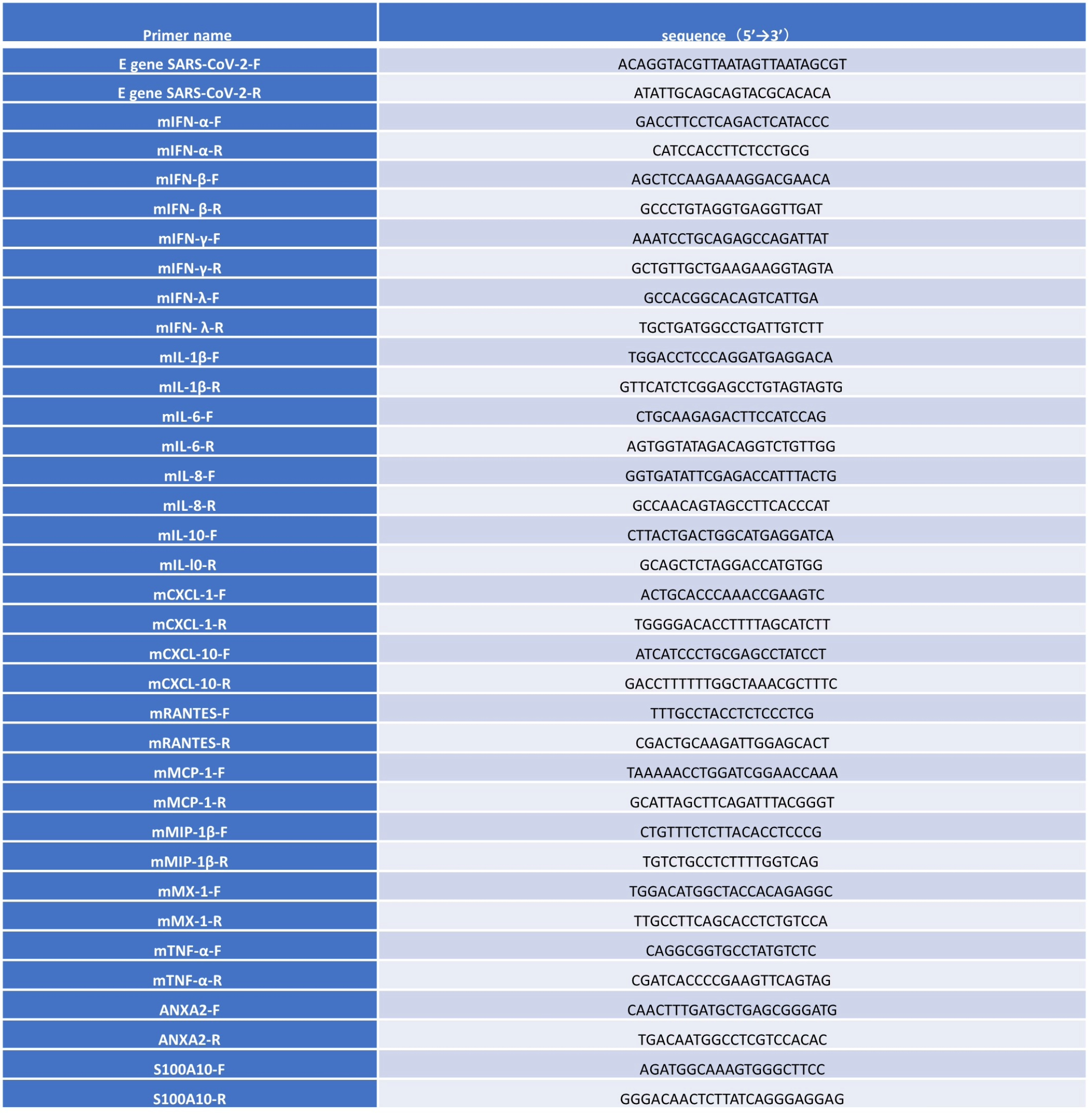

Supplement: S2 Table — (TIF) [file ppat.1011777.s005.tif]
